# Facteurs associés à l’échec du traitement médical de la grossesse extra-utérine: cas de l’Hôpital Gyneco-Obstétrique et Pédiatrique de Yaoundé

**DOI:** 10.11604/pamj.2022.41.200.21175

**Published:** 2022-03-11

**Authors:** Pascal Foumane, Esther Juliette Ngo Um Meka, Félix Essiben, Émeric Lionel Botsomogo, Julius Dohbit Sama, Isidore Tompeen, Etienne Belinga, Emile Mboudou

**Affiliations:** 1 Département de Gynécologie et Obstétrique, Université de Yaoundé I, Yaoundé, Cameroun,; 2 Hopital Gynéco Obstétrique et Pédiatrique de Yaoundé, Yaoundé, Cameroun

**Keywords:** Grossesse extra-utérine, traitement médical, facteurs associés à l’échec, Cameroun, Ectopic pregnancy, medical treatment, factors associated with failure, Cameroon

## Abstract

**Introduction:**

l´objectif de notre travail était d´identifier les facteurs associés à l´échec du traitement médical de la grossesse extra-utérine (GEU) chez les femmes à l´Hôpital Gynéco- Obstétrique et Pédiatrique de Yaoundé.

**Méthodes:**

il s´agissait d´une étude cas-témoins avec collecte rétrospective des données, sur une période de 10 ans, du 1^er^ janvier 2008 au 31 décembre 2017. Les cas étaient toutes les patientes prises en charge pour GEU avec échec du traitement médical et les témoins celles chez qui le traitement médical de la GEU avait réussi. Les variables étudiées étaient: les caractéristiques sociodémographiques, cliniques, paracliniques et thérapeutiques. L´échantillonnage était consécutif et exhaustif. Nous avons réalisé une analyse multivariée.

**Résultats:**

nous avons recruté 92 patientes dont 23 cas et 69 témoins. Les variables associées à l´échec du traitement médical de la GEU après analyse univariée étaient: un taux de β-HCG initial > 10000UI/L (OR=3,05;P=0,031), un taux de β-HCG à J4 > 10000UI/L (OR=7,15;P=0,000), un taux de β-HCG à J7 > à 10000UI/L (OR=20;P=0,000), un score de Fernandez ≥ 13 (OR=3,09;P=0,020), la présence d´activité cardiaque de l´embryon (OR=2,8;P=0,036), l´antécédent d´interruption volontaire de grossesse (OR=2,67;P=0,043) et le niveau de scolarisation primaire (P=0,013). Après analyse multivariée, les facteurs prédictifs étaient: un taux de β-HCG initial >10000 UI/L (OR=8,97; P=0,004), un taux de β-HCG à J4 >10000 UI/L (OR=8,44;P= 0,007), un score de Fernandez ≥ 13 (OR=1,12;P=0,005) et la présence de l´activité cardiaque de l´embryon (OR=6,09;P=0,026).

**Conclusion:**

les facteurs prédictifs de l´échec du traitement médical de la GEU à HGOPY sont liés au taux de β-HCG initial et la viabilité embryonnaire.

## Abstract (en)

**Introduction:**

the purpose of this study was to identify factors associated with the failure of medical treatment for ectopic pregnancy (EP) in women at the Yaoundé Gynaecology, Obstetrics and Pediatrics Hospital.

**Methods:**

we conducted a case-control study using a retrospective data collection over a 10-year period from January 1^st^ 2008 to December 31^st^ 2017. Our study included all patients treated for EP; the study group was composed of patients in whom medical treatment had been unsuccessful while the control group was composed of patients in whom medical treatment had been successful. The variables studied were: socio-demographic, clinical, paraclinical and therapeutic features. Consecutive and complete sampling were used. Multivariate analysis was performed.

**Results:**

we enrolled 92 patients, including 23 cases and 69 controls. The variables associated with the failure of medical treatment for EP after univariate analysis were: initial β-HCG (beta-human chorionic gonadotropin) level > 10000IU/L (OR=3.05; P=0.031), β-HCG level on day 4 > 10000IU/L (OR=7.15;P=0.000), β-HCG level on day 7 > 10000UI/L (OR=20; P=0.000), Fernandez score ≥ 13 (OR=3.09;P=0.020), the presence of fetal heart activity (OR=2.8; P=0.036), a history of voluntary abortion (OR=2.67;P=0.043) and primary level of education. (P=0.013). After multivariate analysis, predictors were: initial β-HCG level>10000 IU/L (OR=8.97; P=0.004), β-HCG level on day 4>10000 IU/L (OR=8.44;P= 0.007), Fernandez score ≥ 13 (OR=1.12;P=0.005), and the presence of fetal heart activity (OR=6.09;P=0,026).

**Conclusion:**

at the Yaoundé Gynaecology, Obstetrics and Pediatrics Hospital predictors of failure of medical treatment for EP were related to initial β-HCG level and fetal viability.

## Introduction

La grossesse extra-utérine (GEU) est l´urgence gynécologique médico-chirurgicale par excellence. Elle correspond à l´implantation et au développement de l´œuf fécondé en dehors de la cavité endométriale [1]. Le diagnostic précoce de la grossesse extra-utérine est possible, incluant les facteurs anamnestiques, que sont les facteurs de risque, les facteurs cliniques (la notion d'aménorrhée), et paracliniques (le dosage quantitatif de l´hormone chorionique gonadotrope (HCG) plasmatique, et l´échographie endovaginale [2]. Ainsi, quand le diagnostic de grossesse extra-utérine est posé précocement, la chirurgie peut être évitée au bénéfice d´une approche médicale conservatrice [2].

L´incidence des grossesses extra-utérines n´a cessé d´augmenter à travers le monde au cours des dernières décennies. Elle représente approximativement 1 à 2% de toutes les grossesses en Occident, soit 10 à 20 grossesses extra-utérines pour 1000 naissances vivantes [3]. Cette incidence est plus élevée dans les pays en voie de développement, particulièrement en Afrique, et au Cameroun en l´occurrence où elle atteint 4,23% [4]. La grossesse extra-utérine a de lourdes conséquences sanitaires et sociologiques. Elle participe activement à la morbidité et à la mortalité liée à la grossesse culminant à 12% selon Tebeu *et al*. en 2005 au Cameroun, tout en étant responsable de stérilité définitive dans 20 à 30% des cas [5]. Vu l´impact sanitaire de cette pathologie récidivante dans 10 à 20% des cas selon Garbin *et al*. en 2010, elle constitue un véritable enjeu de santé publique [6].

Parallèlement aux variations épidémiologiques et à l´amélioration du diagnostic qui est devenu de plus en plus précoce, la prise en charge de la grossesse extra-utérine s´est modifiée en un peu moins d´un demi-siècle. Depuis la publication princeps de Tanaka décrivant une grossesse interstitielle traitée par méthotrexate en 1982, le traitement médical de la grossesse extra-utérine s'est développé et s'est imposé comme une alternative possible au traitement chirurgical dans certaines situations [7]. Aux Etats Unis, Glock *et al*. retrouvent un taux de succès de 85,7% tandis que Foumane *et al*. dans leur étude sur la place du traitement peu ou non invasif dans la prise en charge de la grossesse extra-utérine à l´Hôpital Gynéco-Obstétrique et Pédiatrique de Yaoundé ressortent un taux de 62,71% de succès du traitement médical [4, 8].

Dans la littérature, plusieurs variables sont décrites comme étant corrélées au risque d´échec du traitement médical, notamment le taux élevé initial de l´hormone chorionique gonadotrope humaine, la présence d´une vésicule vitelline à l´échographie et la présence d´un épanchement péritonéal [9, 10]. À notre connaissance, très peu d´études sur les facteurs associés à l´échec du traitement médical de la grossesse extra-utérine ont été réalisées au Cameroun. Les facteurs de risque de survenue de grossesse extra-utérine ainsi que la prise en charge de ces dernières sont les entités les plus rapportées dans les différentes études faites en Afrique [4, 11].

L´objectif de notre travail était d´identifier les facteurs associés à l´échec du traitement médical de la grossesse extra-utérine à l´Hôpital Gynéco-Obstétrique et Pédiatrique de Yaoundé. Il s'agissait spécifiquement de rechercher les variables sociodémographiques présentant une association significative avec l´échec du traitement médical de la grossesse extra-utérine, de déterminer les variables cliniques et paracliniques présentant une association significative avec l´échec du traitement médical de la grossesse extra-utérine. D'identifier les effets secondaires rencontrés au décours du traitement médicamenteux, et de ressortir les modalités du traitement chirurgical en cas d´échec du traitement médical.

## Méthodes

**Conception et cadre de l´étude:** il s´agissait d´une étude cas témoins, réalisée dans le service de gynécologie de l'Hôpital Gynéco-Obstétrique et Pédiatrique de Yaoundé (HGOPY). L'étude s'est étendue sur une période de 10 ans, allant du 1^er^ janvier 2008 au 31 décembre 2017. La collecte des données s´est effectuée de janvier à avril 2018.

**Participants:** l´échantillonnage a été consécutif, non probabiliste et non exhaustif. Notre population cible était constituée de patientes ayant eu une grossesse extra-utérine, éligible au traitement médical, suivie dans le service ou référée à l´HGOPY pendant la période d´étude. Étaient incluses dans l´étude toutes les patientes prises en charge médicalement pour grossesse extra-utérine. Le groupe des cas était constitué des patientes ayant eu un échec du traitement médical de la grossesse extra-utérine. L´échec étant défini par la nécessité de recours à une intervention chirurgicale après réalisation première d´un traitement médicamenteux. Le groupe des témoins quant à lui, était formé des patientes dont le traitement médical avait réussi.

**Taille de l´étude:** pour le calcul de l'échantillon, nous avons utilisé la formule de Schlesselman [12]. Nous avons utilisé les données de Tawfiq *et al*. en 2000 au Canada [9]. La taille de l'échantillon s'élevait donc à 60, soit 15 cas pour 45 témoins, ce qui donne une ration de 1 cas pour 3 témoins. Notre étude a été réalisée dans le respect des principes de la recherche médicale.

**Variables:** après avoir obtenu les autorisations administratives, nous avons procédé à la collecte des données à l'aide d'une fiche technique préconçue, certains sujets ont été joints à travers leur téléphone. Après leur avoir préalablement expliqué les fondements afin d´obtenir leur consentement éclairé, ont accepté de compléter les informations manquantes. Les variables étudiées étaient:

***Les caractéristiques sociodémographiques:*** l´âge en années, le poids, le niveau d´étude, le lieu de résidence, la catégorie professionnelle, le statut matrimonial, la région d´origine.

### Les caractéristiques cliniques

***Les antécédents gynécologiques:*** les ménarches, les anomalies du cycle (dysménorrhées, métrorragies, ménorragies, spanioménorrhées). La notion d´infection sexuellement transmissible, de maladie inflammatoire pelvienne, l´existence de pathologie tubaire avant la conception, l´antécédent de grossesse extra-utérine, d´interruption volontaire de grossesse, de fausse couche spontanée, de manipulation intra-utérine. La notion d´infertilité, la notion de traitement de l´infertilité, le type de contraceptif utilisé

***Les antécédents obstétricaux:*** la gravidité, la parité.

***Les antécédents médico-chirurgicaux:*** notion d´appendicectomie, péritonite, chirurgie sur les annexes.

***Les antécédents toxicologiques:*** la prise de tabac et d´alcool.

**Les caractéristiques cliniques lors du diagnostic:** le motif de consultation, l´aménorrhée en jours, l´existence de métrorragies et de douleurs pelviennes, les éléments du toucher vaginal.

**Les caractéristiques paracliniques:** le taux de β-HCG, l´existence et le résultat du bilan pré-thérapeutique (numération formule sanguine (N.F.S.), dosage des transaminases, créatinémie-urémie, taux de prothrombine-temps de céphaline activée (TP-TCA)), la sérologie VIH, hépatite B, C.

**Les caractéristiques échographiques lors du diagnostic:** l´existence d´un embryon avec activité cardiaque, l´existence et l´abondance de l´hémopéritoine et de l´hématosalpinx, la localisation de la grossesse extra-utérine.

### Le score de Fernandez calculé

**Le traitement médical:** le délai (jours) entre la première injection et la 2^e^, entre la 2^e^ et la 3^e^, le nombre total d´injections de méthotrexate (MTX), l´observance de la surveillance après l´injection, la cinétique des β-HCG et la tolérance du traitement médicamenteux.

**Le traitement chirurgical:** le délai (jour) entre la première injection de MTX et la chirurgie, l´argument principal ayant motivé la réalisation de la chirurgie, la voie d´abord, le geste chirurgical réalisé (salpingotomie/salpingectomie), et l´existence ou non de complications du traitement chirurgical, les trouvailles par opératoire.

**Analyse statistique:** les données ont été saisies à l´aide du logiciel Microsoft Excel 2016. Les résultats ont été analysés avec le logiciel Epi info version 3.5.4 et reportés sous formes de tableaux. Le test statistique khi carré et le test exact de Fisher (quand l´effectif dans une cellule était inférieur à 5) ont été utilisés pour vérifier l´association entre deux variables catégorielles. Seules les valeurs p < 0,05 étaient considérées comme statistiquement significatives. Le degré d´association a été mesuré grâce au calcul du rapport de cotes (Odds ratio = OR) avec leur intervalle de confiance. Une analyse multivariée a été effectuée pour éliminer les facteurs de confusion.

## Résultats

**Participants:** pendant notre étude, 657 grossesses extra-utérines ont été répertoriées dans le registre des archives, mais seulement 329 dossiers ont été retrouvés. Parmi ceux-ci, 230 patientes ont été traitées chirurgicalement en première intention. Les patientes ayant des dossiers incomplets (n=3) n´ont pas été incluses tout comme celle ayant des grossesses hétéro-topiques (n=1). Au total, 92 participantes ont été recrutées, 23 avec échec du traitement médical de la grossesse extra-utérine (cas), qui ont été comparées à 69 femmes avec réussite du même traitement (témoins) ce qui fait un ratio de 1 cas pour 3 témoins.

**Profil sociodémographique:** la moyenne d´âge chez les cas était de 28,39 ans ±4,09 avec les extrêmes de 21 ans et 35 ans, contre 28,96 ans chez les témoins, ± 4,97 avec comme minimum 18 et maximum 45 ans. La tranche d´âge la plus représentée était celle de 20 à 30 ans. Le poids moyen chez les cas était de 70,17 kg ±9,76 avec les extrêmes de 51 kg et 89 kg, contre 70,36 kg chez les témoins, ± 11,77 avec comme minimum 47 et maximum 109 kg. La majorité des patientes était mariée (81,5%), résidait en milieu urbain (96,7%), et étaient élèves ou en autoemploi (26,1%). Les données sociodémographiques sont résumées dans le Tableau 1. Nous n´avons pas trouvé d´association statistiquement significative entre l´âge, le poids, le lieu de résidence, le statut matrimonial, la profession et l´échec du traitement médical de la grossesse extra-utérine. Par contre, nous avons trouvé une association statistiquement typique avec le niveau d´étude primaire (Tableau 1).

**Tableau 1:** variables sociodémographiques associées à l’échec du traitement médical de la grossesse extra-utérine

Variables	Cas n=23(%)	Témoins n=69 (%)	OR*(ICà95%)**	P
**Age (ans)**				
<20	0 (0)	1(1,4)	/	0,559
[20–30]	11 (47,8)	41 (59,4)	0,63(0.24–1,63)	0,332
≥ 30	12 (52,2)	27 (39,1)	1,7(0,66–4,4)	0,273
**Poids (kg)**				
<60	4 (17,4)	12 (17,4)	1 (0,29–3,47)	1,000
[60–80]	14(60,9)	43 (62,3)	0,94 (0,36–2,48)	0,887
≥ 80	5 (21,7)	14 (20,3)	1,09 (0,34–3,45)	0,887
**Zone de résidence**				
Urbain	22 (95,6)	67 (97,1)	0,66 (0,06–7,60)	0,734
Rural	1 (4,4)	2 (2,9)	1,53(0,13–17,59)	0,734
**Statut matrimonial**				
Mariée	20 (86,9)	55 (79,7)	1,7 (0,44–6,54)	0,438
Célibataire	3 (13,1)	14 (20,3)	0,59 (0,15–2,27)	0,438
**Niveau de scolarisation**				
Primaire	2 (8,7)	0 (0,0)	**/**	**0,013**
Secondaire	6 (26,1)	17 (24,6)	1,08 (0,37–3,18)	0,887
Supérieur	15 (65,2)	52 (75,4)	0,61 (0,22–1,69	0,342
**Profession**				
Fonctionnaire	2 (8,7)	12 (17,4)	0,45 (0,09–2,18)	0,314
Salariée du secteur privé	4 (17,4)	8 (11,6)	1,45 (0,39–5,37)	0,577
Elève/étudiante	6 (26,1)	14 (20,3)	1,39 (0,46–4,18)	0,559
Auto–emploi	6 (26,1)	26 (37,7)	0,58 (0,2–1,66)	0,312
Ménagère	5 (21,7)	9 (13)	1,85 (0,55–6,23)	0.314

* OR: Odds Ratio **IC (95%): Intervalle de Confiance à 95%, P: valeur P

**Variables cliniques:** l´analyse des variables cliniques nous a permis d'identifier l´antécédent d´interruption volontaire de grossesse (IVG) comme facteur associé à l´échec du traitement médical de la grossesse extra-utérine (OR = 2,67; P=0,043).

**Variables paracliniques:** les variables paracliniques associées à l´échec du traitement médical de la grossesse extra-utérine était les suivantes: un taux de β-HCG >10000 UI/L à J0 (OR=3,05; P=0,031), à J4 (OR=7,15; P=0,000), un score de Fernandez supérieur à 13 (OR =3,09; P=0,020). La présence d´activité cardiaque chez l´embryon a été identifié comme facteurs associés à l´échec du traitement médical de la grossesse extra-utérine (OR=2,8; P=0,036) (Tableau 2). Par contre, le taux de β-HCG entre 0 et 1500 UI/L, était retrouvé comme facteur protecteur à J0 (OR=0,15; P=0,006), à J4 (OR=0,16; P=0,014), à J7 (OR=20; P=0,000) de même que le score de Fernandez inférieur à 13 (OR=0,32; P=0,020) (Tableau 2).

**Tableau 2:** tableau récapitulatif des variables significatives associées à l’échec du traitement médical de la grossesse extra-utérine, après analyse univariée

Variables	Cas n=23 (%)	Témoins n=69 (%)	OR*(IC 95%)**	P
Niveau de Scolarisation Primaire	2 (8,7)	0 (0,0)	/	0,013
Antécédent d’IVG	12 (52,1)	20 (28,9)	2,67 (1,01–7,04)	0,043
β–HCG J0 >10000	9 (39,1)	12 (8,7)	3,05 (1,07–8,66)	0,031
β–HCG J4 >10000	11 (64,70)	10 (20,41)	7,15 (2,13–24,06)	0,000
β–HCG J7 >10000	6 (63,4)	2 (9,9)	20 (2,68–149,03)	0,000
Score de Fernandez ≥13	12 (52,18)	18 (26,08)	3,09 (1,16–8,22)	0,020
Présence activité cardiaque embryon	11(47,8)	17 (20,28)	2,8 (1,05–7,49)	0,036

**Analyse multivariée:** après analyse multivariée, les facteurs prédictifs d'échec étaient: un taux de β-HCG initial >10000 UI/L (OR=8,97; P=0,004), un taux de β-HCG à J4 > 10000 UI/L (OR=8,44; P= 0,007), un score de Fernandez ≥ 13 (OR=1,12; P=0,005) et la présence de l´activité cardiaque de l´embryon (OR=6,09; P=0,026) (Tableau 3)

**Tableau 3:** variables significatives associées à l’échec du traitement médical de la grossesse extra-utérine, après analyse multivariée et régression logistique

Variables	OR* ajusté	IC (95%)	P
Antécédent d'IVG	3,04	0,48–6,05	0,109
β–HCG J0 >10000	8,97	1,94–14,8	0,004
β–HCG J4 >10000	8,44	3,54–8,70	0,007
β–HCG J7 >10000	6,21	0,78–9,14	0,073
Score de Fernandez ≥13	1,12	1,05–3,59	0,005
Présence de l'activité cardiaque de l'embryon	6,09	3,25–9,56	0,026

## Discussion

**Limites de l´étude:** la nature rétrospective de la collecte des données expose à un biais de sélection, car les dossiers médicaux n´étaient pas toujours complets. En outre, nous n´avons pas fait d´appariement entre les patientes au début de l´étude par soucis de recenser tous les facteurs de risque. Afin d´éliminer les facteurs de confusion liés à ce défaut d´appariement, nous avons effectué une analyse multivariée par régression logistique. Enfin, les patientes considérées comme ayant réussi le traitement médical ont, pour la plupart, arrêté la surveillance avant d´atteindre la négativation complète des β-HCG.

**Les données sociodémographiques:** l´antécédent d´interruption volontaire de grossesse a été retrouvé comme statistiquement associé à l´échec du traitement médical de la grossesse extra-utérine. Aussi, il avait été identifié comme facteur de risque de grossesse extra-utérine dans les pays en voie de développement par Anorlu *et al*. en 2005 au Nigéria [13]. Bien que l´antécédent de grossesse extra-utérine ait été identifié comme facteur associé à l´échec du traitement médical de la grossesse extra-utérine par Lipscomb *et al*. en 2004, et que l´interruption volontaire de grossesse soit l´une de ses étiologies dans notre contexte, ceci n'a pas été confirmé après analyse multivariée [14].

**Le niveau de scolarisation:** nous avons retrouvé une association statistiquement significative avec un niveau d´instruction primaire. En effet, un faible niveau d´instruction conduit souvent à des emplois qui ne requièrent pas un grand niveau de compétences, par conséquent associés à un faible revenu. Ceci réduit généralement l´accès aux soins, et donc une prise en charge habituellement tardive pouvant être à l´origine de l´échec thérapeutique médical de la grossesse extra-utérine. En outre, Agholor *et al*. en 2013 au Nigéria ont identifié le niveau d´instruction primaire comme un facteur de risque de grossesse extra-utérine avec un OR=6,32 [15].

### Les données cliniques

**La douleur pelvienne:** Tawfiq *et al*. en 2000 ont démontré que la présence d´une douleur pelvienne au moment de la consultation entrainait un taux d´échec de 56% contre 17% sans douleur, ceci avec un Odds ratio = 9,20 [9]. Ceci pouvant être interprété comme un signe d´envahissement de la séreuse tubaire ou de pré-rupture, limitant ainsi l´action du méthotrexate. Dans notre étude, nous avons retrouvé une association entre la douleur spontanée et l´échec du traitement médical, mais elle n´était pas statistiquement significative. Nous pensons que cette discordance pourrait être d´ordre méthodologique, notamment liée à la taille de notre échantillon.

**Les données paracliniques:** un taux de β-HCG supérieur à 10000 UI/l à J0 s´est révélé être un facteur associé statistiquement typique de l´échec du traitement médical de la grossesse extra-utérine dans notre étude. Ces résultats sont en accord avec ceux de Lipscomb *et al*. en 1999 qui ont affirmé que le facteur majeur de l´échec du traitement médical de la GEU était le taux élevé initial de β-HCG [16]. En Europe, Corsan *et al*. ont identifié un taux supérieur à 1500 UI/L comme associé à l´échec du traitement médical tandis que Dilbaz *et al*. aux États-Unis retrouvaient un taux supérieur à 3000 UI/L [17, 18]. Nos résultats diffèrent de ceux de Menon *et al*. et Mboudou *et al*. en 2007, qui ont aussi révélé comme facteurs influençant la réussite un taux de β-HCG élevé, mais à partir de 5000 UI/L [19, 20]. Cette différence pourrait être due à la non-limitation du taux de β-HCG dans nos critères d´inclusion. Le point de discussion est la valeur maximale admissible du taux de β-HCG. En effet, le taux élevé de β-HCG est un élément de mauvais pronostic, rendant le succès incertain. En revanche, il est remarquable de constater que la plus grande série américaine n'intègre pas les dosages hormonaux pour décider de l'opportunité d'un traitement par MTX [21]. Il semble cependant souhaitable d'informer les patientes que l'existence d´un taux de β-HCG supérieur à 5000 UI/L est corrélée à l'utilisation plus fréquente d'une deuxième dose de MTX et à un délai de négativation plus long de β-HCG, rendant ainsi le suivi post-thérapeutique plus astreignant encore [22].

Dans notre étude, un taux de β-HCG à J4 >10000 UI/l s´est révélé être un facteur associé statistiquement significatif de l´échec du traitement médical de la grossesse extra-utérine. En outre, la moyenne du taux de β-HCG à J4 est plus élevée dans le groupe des échecs que dans celui du succès (15547,4 contre 6049,82 UI/L). Ces résultats sont similaires à ceux de Girija *et al*. en 2017, qui avaient retrouvé un taux moyen de β-HCG à J4 significativement supérieur dans le groupe des échecs [23]. Plusieurs études ont démontré qu´une chute du taux de β-HCG au quatrième jour post méthotrexate prédisait un taux de succès élevé, tel que Nguyen *et al*. en 2010 qui avaient obtenu 100% de réussite chez les patientes ayant connu cette baisse à J4 [23-27].

Par déduction, nous pouvons déduire que la persistance de taux élevés de β-HCG à J4 est un élément de mauvais pronostic, donc associé à l´échec du traitement médical de la GEU. Dilbaz *et al*. en 2006 ont retrouvé un taux supérieur ou égale à 3500 UI/L à J3 comme significativement prédictif de l´échec du traitement (OR=42,9; CI=4,3-421) [18]. Cette différence pourrait s´expliquer par les taux élevés initiaux inclus dans notre étude et la mesure précoce (J3) du taux de β-HCG de contrôle. Par contre, Gabbur *et al*. en 2006 ont affirmé que le taux de β-HCG à J4 ne pouvait être pris en compte pour prédire l´issue du traitement médical à dose unique [28]. Ceci pourrait s´expliquer par une décroissance plus lente des β-HCG pour un protocole unidose.

Le taux de β-HCG traduit l´évolutivité de la GEU, sa cinétique permet au clinicien d´apprécier l´efficacité du traitement. Une mauvaise décroissance ou une non-décroissance du taux de β-HCG à J4 est très souvent retrouvé lors de l´échec du traitement médical de la grossesse extra-utérine. Pour ce qui est des taux de β-HCG sériques à J7, avons retrouvé un taux de β-HCG à J7 > 10000 UI/L comme facteur associé statistiquement significatif de l´échec du traitement médical de la grossesse extra-utérine. Ces résultats vont dans le même sens que ceux de Dudley *et al*. en 2004 qui ont affirmé que l´augmentation permanente du taux de β-HCG post méthotrexate est un facteur indépendant de rupture tubaire tandis que Kirk *et al*. en en 2007 aux Etats Unis ont retrouvé qu´une baisse de 15% du taux de β-HCG entre J4 et J7 entrainait un taux de réussite 90,3% [29, 24]. Ainsi, nous pouvons penser que la persistance de taux très élevés de β-HCG une semaine après l´injection de méthotrexate est un élément en faveur de l´échec du traitement médical de la GEU. D´ailleurs, des taux élevés de β-HCG signent une très grande activité trophoblastique, néfaste à la régression de la GEU après injection de méthotrexate. Par contre Çelik *et al*. en 2013 au terme de leur étude évaluant les variations du taux de β-HCG entre J0-J1, J0-J4 et J0-J7, ont affirmé que le meilleur prédicteur de l´issue du traitement médical était la variation du taux de β-HCG de J0 à J4 avec un taux de succès de 89,6% en cas de baisse [30].

La présence de l´activité cardiaque de l´embryon, dans plusieurs études, était fortement associée à l´échec du traitement médical de grossesse extra-utérine [31, 16]. Nous avons également retrouvé une association statistiquement significative entre cette variable et l´échec du traitement médical. L´activité cardiaque de l´embryon perçue à partir de la 7^e^ semaine traduit une grossesse extra-utérine avancée, souvent de mauvais pronostic pour un traitement médical. Néanmoins, Lipscomb *et al*. en 1998 ont obtenu un taux de succès de 87,5% chez des patientes avec activité cardiaque de l´embryon, soutenant ainsi l´idée qu´elle ne constitue qu´une contre-indication relative au traitement médical de la grossesse extra-utérine [21].

En ce qui concerne le score de Fernandez, notre étude a permis de mettre en exergue une association statistiquement typique entre un score de Fernandez ≥ 13 et l´échec du traitement, tandis qu´un score < 13 s´est révélé protecteur. En effet, 50% des patientes avec échec du traitement avaient un score ≥ 13. Ces résultats sont similaires à ceux de Fernandez H *et al*. en 1991 qui ont obtenu un taux de 50% d´échec du traitement médical pour un score ≥ à 13, contrastant avec un taux de réussite de 82% pour un score < à 13 [32]. Etant donné que le score de Fernandez englobe plusieurs paramètres cliniques et paracliniques, il permet d´orienter le choix du mode de prise en charge efficacement: C´est donc un excellent facteur prédictif de l´échec du traitement médical. Néanmoins, du fait de la progestéronémie rarement réalisée dans notre contexte, il pourrait constituer un biais dans le choix du mode de prise en charge des grossesses extra-utérines. Argument principal ayant motivé la chirurgie: les arguments principaux retrouvés lors de notre étude sont la persistance de la douleur et la non-décroissance des β-HCG (39,1%). Ils sont similaires à ceux de Tawfiq *et al*. en 2010 et de Potter *et al*. en 2003, qui avaient eux aussi identifié la persistance de la douleur et une mauvaise cinétique des β-HCG comme arguments principaux [11, 33]. En effet, la persistance de cette symptomatologie est très souvent interprétée comme des signes avant-coureurs de pré-rupture tubaire.

Voie d´abord et geste effectué: 18 patientes soit 78,26% ont bénéficié d´une laparotomie contre 5 (21,74%) d´une laparoscopie. Ces résultats sont superposables à ceux de Foumane *et al*. en 2011 dans le même environnement qui avaient obtenus un taux de 79% de laparotomie [4]. La salpingectomie a été réalisé chez 91,30% (21) des patientes. Nayama *et al*. en 2005 au Niger ont pratiqué une chirurgie radicale dans 87,4% des cas, tendance retrouvée dans notre étude. Un taux si élevé de salpingectomie et de laparotomie pourrait s´expliquer par des équipements fréquemment en panne, un personnel insuffisant et un diagnostic tardif lorsque l´instabilité hémodynamique s´est installée, tel que constaté par Foumane *et al*. [4, 34]. Néanmoins, il contraste avec les taux élevés du traitement conservateur retrouvés en occident [35].

## Conclusion

Les facteurs restés associés à l´échec du traitement médical de la grossesse extra-utérine étaient: un taux de β-HCG initial > 10000 UI/L; un taux de β-HCG à J4 > 10000UI/L; un score de Fernandez ≥ 13 et la présence d´activité cardiaque de l´embryon.

### Etat des connaissances sur le sujet

- L´incidence des grossesses extra-utérines n´a cessé d´augmenter à travers le monde au cours des dernières décennies;
- Cette incidence était de 4,23% au Cameroun en 2010;
- Très peu d´études sur les facteurs associés à l´échec du traitement médical de la grossesse extra-utérine ont été réalisées au Cameroun.

### Contribution de notre étude à la connaissance

- Les facteurs trouvés associés à l´échec du traitement médical de la grossesse extra-utérine sont: un taux de β-HCG initial > 10000 UI/L, un taux de β-HCG à J4 > 10000UI/L, un score de Fernandez ≥ 13, La présence d´activité cardiaque de l´embryon.
